# 

**Published:** 1878-04

**Authors:** 


### Contents of April Number.

### ORIGINAL.

ART. I.—Morbus Coxarius. By E. R. Barnes, M. D.,................ 321
ART. II.—Intussusception. By A. A. Hubbell, M. D.,............... 328
ART. III.—Proceedings of the Buffalo Medical Association. Case of Perityphlitic Abcess. By Prof. Rochester,.................... 332
ART. IV.—A Model Pocket Case. By C. C. F. Gay, M. D.,.......... 334

### CORRESPONDENCE.

Hot Springs. Ethics or Quackery, which? By Charles A. Wall, B. S.,. 336
Homœopathic Medical School in Buffalo, .. ..................... ....
Medical Witnesses shall receive compensation as experts. Prof. J. P. White, M. D.,............................................. 339

### MISCELLANEOUS.

Cases of Rheumatic Fever Treated by Salicylic Acid, 341. Bicarbonate of Soda Dressings for Burns, 345. Conclusions in regard to Gastrotomy, 345. Temperature in Croup, 346. Professional overwork, 347. Suspension in Spinal Disease, 347. Symptoms of Intestinal Obstruction, 348. Treatment of Sleeplessness, 348. A Daring Therapeutist, 349. Cascara Sagrado in Constipation, 349. Excision of the Larynx, 350. Nitrite of Amyl, 350. Cure of Epilepsy, 350. Extract of Pimento as a Revulsive, 350. Action of Morphia taken after Quinine, 351.

### EDITORIAL.

American Medical Association. Surgical Section,................. .... 351
Medical Notes and Comments. By Charles A. Wall, B. S.,............ 352
Books Reviewed:
Materia Medica. Biddle, .... ................................ 356
Action of Medicine. Ott,.......................................... 357
Transactions of the Medical Society of Pennsylvania,.......... 357
A Guide to Therapeutics and Materia Medica. Farquharson,.. 358
Gonorrhœa and Syphilis. Durkee,......... ................. 359
Books and Pamphlets Received,......................... ........ 359

